# Variational iteration method for the nanobeams-based N/MEMS system

**DOI:** 10.1016/j.mex.2023.102465

**Published:** 2023-10-28

**Authors:** Wei Tang, Naveed Anjum, Ji-Huan He

**Affiliations:** a Suzhou International Foreign Language School, Suzhou 215131, China; b Department of Mathematics, Government College University, Faisalabad, Pakistan; c National Engineering Laboratory for Modern Silk, College of Textile and Engineering, Soochow University, Suzhou, PR China

**Keywords:** N/MEMS, Nonlinear oscillator, Elzaki transform, Variational iteration method, Amplitude-frequency relationship, Elzaki transform-based variational iteration method

## Abstract

The nano/microelectromechanical system (N/MEMS) has triggered worldwide concern, and its applications have revolutionized technologies in various advanced fields from wearable sensors, 5 G communication technology, to energy harvesting, to aerospace. However, when the applied force is sufficiently large, the pull-in instability arises, and reliable operation is forbidden. Therefore, it is extremely important to insight fast and accurately into the periodic motion of the system to prevent the system from its pull-in motion. The basic aim of this study is to demonstrate the applicability of the well-known variational iteration method (VIM) for predicting the dynamic behavior of N/MEMS. For this, a nanobeam-based microstructure with van der Waals force for actuation is used as an example to reveal its periodic properties. The governing equation for the oscillation of the microsystem is obtained from the Euler-Bernoulli beam principle, considering the midplane stretching effect. We then employ the Galerkin technique to transform the governing partial differential equation into an ordinary differential equation, which is highly nonlinear, making it extremely difficult to solve by some traditional analytical methods, however, the VIM shows its ability to elucidate accurately the basic properties of the N/MEMS by simple calculation. This paper offers a new road for fast and accurate prediction of the microsystem's properties, and the result can be used for optimizing the N/MEMS.

• A nanobeam-based N/MEMS system with van der Waals force is considered.
• A strongly governing equation without a linear term is obtained.
• The variational iteration method is applied to figure out the basic properties of the system.

## Graphical abstract

**Figure:**
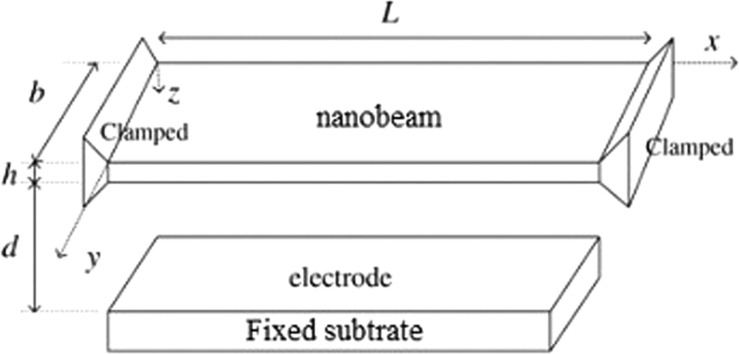

Specifications table

**Table:** 

Subject area:	Mathematics and Statistics
More specific subject area:	Study of the dynamics of the nano/microelectromechanical systems actuated by van der Waals forces
Name of your method:	Elzaki transform-based variational iteration method
Name and reference of original method:	Variational Iteration Method (VIM), ref: 15. J.H. He, Variational iteration method – a kind of non-linear analytical technique: some examples, Int J Non Linear Mech 34 (1999) 699–708
Resource availability:	Anjum N, Suleman M, Lu D, He JH, Ramzan M, Numerical iteration for nonlinear oscillators by Elzaki transform, J. Low Freq. Noise Vib. Act, 2019, 39 (4), 879–884.

## Method details

The nano/microelectromechanical system (N/MEMS) is an innovative new technology with outstanding properties in geometrical size, reliable operation and high sensitivity. The system achieves this feat of benign properties by judiciously controlling the applied voltage. The system is composed of both stationary and movable components. The moving part bends toward the fixed-part when an actuation force is applied, causing the system to perform the periodic motion. These fixed-moveable microstructures have several potential applications in different fields ranging from wearable sensors, 5 G communication technology, imaging process, mechanical sensing, energy harvesting to aerospace [1,2], and its applications have revolutionized technologies in the applied fields [3], and mechanicians have been trying to elucidate its solution properties [4], [5], [6].

N/MEMS devices typically have a small size, frequently just at the micron level, and may not be larger than one centimeter. The modern integrated circuit technology sector can produce thousands of small gadgets with increased efficiency and lower cost due to this high surface-to-volume ratio. However, numerous factors such as actuation force, large deflection, and geometry of the micro-instruments make these structures nonlinear. If these computational challenges are ignored, the nonlinearity in N/MEMS may result in inaccurate judgment.

There are various remarkable methods proposed for N/MEMS, for example, He's frequency formulation [7,8] which is known by its simplicity, the homotopy perturbation method [9] that can handle nonlinear systems, where energy dissipation or external forcing is present, making it applicable to a broader range of practical problems, the iteration perturbation method [10,11] used to study the sensitivity of the solutions of the problems with small perturbation parameters, Adomian decomposition method [12] which typically converges rapidly for the problems with variable coefficients and mixed-order derivatives, Hamiltonian approach [13,14] that allows a deeper understanding of the system's behavior by keeping the total energy of the system constant, and many others. However, each of these approaches has its own set of drawbacks. Discretization of the nonlinear terms, handling perturbation parameter, sensitivity of the initial conditions, nonguaranteed convergence for chaotic systems, handling discontinuities or singularities of the systems are all examples of drawbacks. In this paper, we eliminate the aforementioned drawbacks and suggest the variational iteration method (VIM) [15] coupled with an integral transform from the family of Laplace transform that offers tremendous potential for accurately resolving.

The variational iteration method [15] is recognized as an effective method for solving linear, nonlinear, initial value, boundary value equations, see for examples Refs. [16], [17], [18], [19]. One of the most attractive features of VIM over other analytical methods is that it is unnecessary to linearize or resolve the nonlinear terms. By employing an appropriate initial guess and incorporating Lagrange multipliers, it is possible to obtain exact or highly precise outcomes for both linear as well as nonlinear problems. However, it is not easy to identify the multiplier without the knowledge of the elusive theory of variational calculus [6]. This brief research report proposes an easier way to find the multiplier, making the technique accessible to engineers, physicists, and scientists facing nonlinear problems of N/MEMS excited by van der Waals forces. Only a single iteration is required to attain favorable results with this modification.

This research considers the nonlinear dynamic oscillatory behavior of doubly clamped nanobeam actuated by van der Waals forces. The non-dimensional form of the mathematical model is obtained by utilizing Galerkin's approximation considering the mid-plane stretching effect. Then VIM is paired with the Elzaki transform (EVIM) [20] to produce the desired system solution. The EVIM results are compared to those produced using the 4th-order Runge-Kutta method (RK4). To ensure the EVIM's efficiency, we compare its outcomes to those indicated in Ref. [21] using the spreading residue harmonic balance method (SRHBM).

Recently Anjum and He [22] observed the fact that the correction functional in VIM with an integral transform is extremely suitable for a nonlinear oscillator. The modified variational iteration method was called as the Laplace based variational iteration method [23] or He-Laplace method [24] in literature.

Let's illustrate briefly the idea of VIM used with the Elzaki transform [20]. Assume the general form of the nonlinear oscillatory equation:

(1) $$\ddot{x}\left(t\right)+f\left(x\right)=0$$

Eq. (1) can be rewritten in the following form

(2) $$\ddot{x}+\omega^{2}x+g\left(x\right)=0$$

where ω is the frequency of the oscillatory system to be derived. Moreover, $g\left(x\right)=f\left(x\right)-\omega^{2}x$.

Employing the basic concept of VIM, the iterative formula for Eq. (2) is given as

(3) $$x_{k+1}\left(t\right)=x_{k}\left(t\right)+\int_{0}^{t}\lambda \left(\psi \right)\left[{\ddot{x}}_{k}\left(\psi \right)+\omega^{2}x_{k}\left(\psi \right)+{\tilde{g}}_{k}\left(x\right)\right]\;d\psi ,\;\;k=0,\;1,\;2,\;3,\ldots \ldots$$

where ${\tilde{g}}_{k}$ is a restricted variation i.e., $\delta {\tilde{g}}_{k}=0,$ $x_{k}$ is the *k*th approximate solution and λis the Lagrange multiplier. Since the integral in Eq. (3) is primarily a convolution, the Elzaki transform [20] can be employed by taking the Lagrange multiplier as $\lambda =\bar{\lambda }\left(t-\psi \right)$ in [19].

$$E\left[x_{k+1}\left(t\right)\right]=E\left[x_{k}\left(t\right)\right]+E\left[\int_{0}^{t}\lambda \left(t-\psi \right)\left[{\ddot{x}}_{k}\left(\psi \right)+\omega^{2}x_{k}\left(\psi \right)+{\tilde{g}}_{k}\left(x\right)\right]d\psi \right],\;\;k=0,\;1,\;2,\;\ldots \ldots$$

(4) $$E\left[x_{k+1}\left(t\right)\right]=E\left[x_{k}\left(t\right)\right]+\frac{1}{v}E\left[\lambda (t)\right]E\left[{\ddot{x}}_{k}\left(t\right)+\omega^{2}x_{k}\left(t\right)+{\tilde{g}}_{k}\left(x\right)\right],\;\;k=0,\;1,\;2,\;\ldots \ldots$$

where *v* is the transformed variable. Utilizing the Elzaki transform [20] on above equation and its stationary condition leads to the result

(5) $$\lambda \left(t\right)=-\frac{1}{\omega }\sin\omega t$$

Thus, using Eq. (4), the iteration algorithm takes the following formulation:

$$E\left[x_{k+1}\left(t\right)\right]=E\left[x_{k}\left(t\right)\right]-\frac{1}{\omega }E\left[\int_{0}^{t}\sin\omega \left(t-\psi \right)\left[{\ddot{x}}_{k}\left(\psi \right)+\omega^{2}x_{k}\left(\psi \right)+{\tilde{g}}_{k}\left(x\right)\right]\;d\psi \right]$$

(6) $$x_{k+1}\left(t\right)=x_{k}\left(t\right)-\frac{1}{\omega }E^{-1}\left(\frac{1}{v}E\left[\sin\omega t\right]E\left[{\ddot{x}}_{k}\left(\psi \right)+\omega^{2}x_{k}\left(\psi \right)+{\tilde{g}}_{k}\left(x\right)\right]\right)$$

For more details about the method, readers can see the Ref. [20].

As the EVIM is based on the Taylor series expansion and the subjected problem is of oscillatory nature, conventional convergence criteria may not adequately capture the behavior of the technique. The convergence criteria in this case can be influenced by various factors, such as amplitude error, phase error, or frequency deviation. To meet the substantial criteria, one should optimally choose the initial guess, Lagrange multiplier and the discretization of the domain.

## Model problem formulation

The nanobeams are the major structural elements and play a vital role in N/MEMS. Beam theories are mathematical frameworks that make it easier to analyses beam behavior. These theories offer a way to understand and predict the deformation and stress distribution within beams subjected to various loads. There are several beam theories, like Euler-Bernoulli, Timoshenko, Bernoulli-Rayleigh, Vlasov, Reissner-Mindlin, Reddy etc. As our problem is of nano scale, Euler-Bernoulli beam theory [25] is employed in this paper which is best suitable for analyzing beams with relatively small deformations compared to their length.

**Fig. 1:**
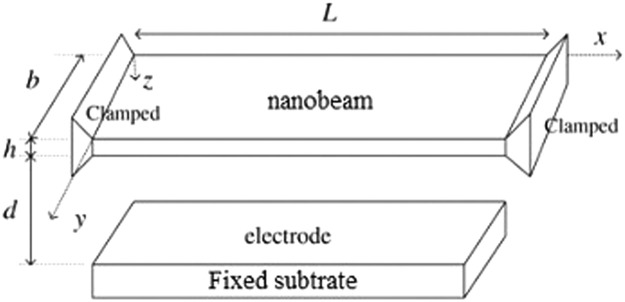
Doubly clamped nanobeam based N/MEMS.

Assume a clamped-clamped nanobeam [26] with length L, width b, thickness h and density ρ as shown in Fig. 1. The governing equation for nanobeam deflection can be generated using Newton's Law, which depends on the Euler-Bernoulli beam concept and is stated as:

(7) $$EI\frac{\partial^{4}W}{\partial \chi^{4}}+\rho S\frac{\partial^{2}W}{\partial \tau^{2}}-\left[\tilde{N}+\frac{ES}{2L}{\int_{0}^{L}\left(\frac{\partial W}{\partial \chi }\right)}^{2}\;d\chi \right]\frac{\partial^{2}W}{\partial \chi^{2}}-F\left(\chi ,\tau \right)=0$$

where W(χ,τ) is a function of χ(location) and τ(time), demonstrating the mid-plane deflection of nanobeam, E, $I=bh^{3}/12$, S=bh and $\tilde{N}$ are, respec tively, the Young's modulus, moment of inertia of nanobeam about Y axis, the cross-section area and the axial load, F(χ,τ) is the van der Waals force between the nanobeam and substrate, which can be expressed as [25]:

(8) $$F\left(\chi ,\tau \right)=\frac{A_{h}b}{6\pi {\left(d-W\right)}^{3}}$$

where $A_{h}$ denotes the Hamaker coefficient, $30\times 10^{-19}J<A_{h}<50\times 10^{-19}J$ and d is the distance between the nanobeam and its substrate. The conditions on boundaries of the system is of clamped-clamped nature and can be stated:

(9) $$W\left(0,\tau \right)=W\left(L,\tau \right)=0,\;\;{\frac{\partial W}{\partial \chi }|}_{\left(0,\tau \right)}={\frac{\partial W}{\partial \chi }|}_{\left(L,\tau \right)}=0$$

For simplicity, the non-dimensional form of the space, deflection and time variables can respectively be chosen as:

(10) $$\eta =\frac{\chi }{L},\;\;\;\;\;\;\;\;\;\;\;w=\frac{W}{d},\;\;\;\;\;\;\;\;\;\;\;t=\frac{\tau }{\tilde{T}},$$

where

(11) $$\tilde{T}=\sqrt{\frac{\rho hbL^{4}}{EI}}$$

After simple operations, Eq. (7) leads to the following dimensionless form:

(12) $$\frac{\partial^{4}w}{\partial \eta^{4}}+\frac{\partial^{2}w}{\partial t^{2}}-\left[N+\alpha {\int_{0}^{1}\left(\frac{\partial w}{\partial \eta }\right)}^{2}\;d\eta \right]\frac{\partial^{2}w}{\partial \eta^{2}}-\frac{\lambda }{{\left(1-w\right)}^{3}}=0$$

where the non-dimensional parameters N(axial load), α(aspect ratio) and λ(van der Waals force) in Eq. (12) are:

(13) $$N=\frac{\tilde{N}L^{2}}{EI},\;\;\;\;\;\;\;\;\;\;\;\alpha =6{\left(\frac{d}{h}\right)}^{2},\;\;\;\;\;\;\;\;\;\;\;\lambda =\frac{12A_{h}}{Eh^{3}}{\left(\frac{L}{d}\right)}^{4}$$

Dimensionless boundary constraints can also be represented as:

(14) $$w\left(0,t\right)=w\left(1,t\right)=0,\;\;{\frac{\partial w}{\partial \eta }|}_{\left(0,\tau \right)}={\frac{\partial w}{\partial \eta }|}_{\left(1,\tau \right)}=0$$

The governing equation established here provides a broad description of the periodicity property of the nanobeam, however, the singularity when w=1 in Eq. (12) makes it difficult to be solved. He & Liu suggested a variational approach to a singular wave [27]. Here the Galerkin approach is applied to reduce Eq. (12) into an ordinary differential equation. For this, the deflection function w(η,t) can be expressed as the product of two separate functions as:

(15) w(η,t)=ξ(η)x(t)

where x(t) and ξ(η) are further to be determined. Here, we choose ξ(η)in the form [28]:

(16) $$\xi \left(\eta \right)=16\eta^{2}{\left(1-\eta \right)}^{2}$$

Eq. (15) can be substituted in Eq. (12), and the governing equation is then multiplied by $\xi \left(\eta \right){\left(1-w\right)}^{3}$ and integrated across dimensionless domain, and we obtain

(17) $$\begin{array}{ccc} & & \int_{0}^{1}\xi {\left(1-\xi x\right)}^{3}x{\xi^{\prime \prime \prime }}^{\prime }\;d\eta +\int_{0}^{1}\xi^{2}{\left(1-\xi x\right)}^{3}\ddot{x}\;d\eta \\ & & -\;\int_{0}^{1}\xi {\left(1-\xi x\right)}^{3}\left(N+\alpha {\int_{0}^{1}\left(\frac{\partial w}{\partial \eta }\right)}^{2}\;d\eta \right)x\xi^{\prime \prime }\;d\eta -\int_{0}^{1}\lambda \xi d\eta =0 \end{array}$$

where the dot denotes the derivative with respect to t and the prime signifies the derivative with respect to η. Now, we can write Eq. (17) as:

(18) $$\left(h_{0}+h_{1}x+h_{2}x^{2}+h_{3}x^{3}\right)\ddot{x}+h_{4}+h_{5}x+h_{6}x^{2}+h_{7}x^{3}+h_{8}x^{4}+h_{9}x^{5}+h_{10}x^{6}=0$$

where the expressions for $h_{j}\;\left(j=0∼10\right)$ are given in Appendix A. The initial conditions of Eq. (18) are given as follows

(19) $$x\left(0\right)=B,\;\;\dot{x}\left(0\right)=0$$

Eq. (18) is highly nonlinear, so it is not so easy to be solved by some famous analytical methods [29], [30], [31], [32], this offers the variational iteration method a new chance in a hunt for exact prediction of the periodic property of Eq. (18) with the initial conditions of Eq. (19).

## Application of method of solution for vibrations of nanobeam

This section focuses on using EVIM to discover an estimated solution to the model being studied. For this, Eq. (18) can be written as

(20) $$\left(1+d_{1}x+d_{2}x^{2}+d_{3}x^{3}\right)\ddot{x}+d_{4}+d_{5}x+d_{6}x^{2}+d_{7}x^{3}+d_{8}x^{4}+d_{9}x^{5}+d_{10}x^{6}=0$$

where $d_{j}=\frac{h_{j}}{h_{0}}$ for j=1,2,⋯,10. We can express Eq. (20) as

(21) $$\ddot{x}+\omega^{2}x+g\left(x\right)=0$$

where $g\left(x\right)=\left(d_{1}x+d_{2}x^{2}+d_{3}x^{3}\right)\ddot{x}+d_{4}+\left(d_{5}-\omega^{2}\right)x+d_{6}x^{2}+d_{7}x^{3}+d_{8}x^{4}+d_{9}x^{5}+d_{10}x^{6}$

With the aid of Eq. (16), it is possible to write the iterative formula for Eq. (21) as:

(22) $$x_{k+1}\left(t\right)=x_{k}\left(t\right)-\frac{1}{\omega }E^{-1}\left(\frac{1}{v}E\left[\sin\omega t\right]E\left[\left(1+d_{1}x_{k}+d_{2}x_{k}^{2}+d_{3}x_{k}^{3}\right)\ddot{x}+d_{4}+d_{5}x_{k}+d_{6}x_{k}^{2}+d_{7}x_{k}^{3}+d_{8}x_{k}^{4}+d_{9}x_{k}^{5}+d_{10}x_{k}^{6}\right]\right)$$

In the light of initial conditions given in Eq. (19), we can assume the initial guess as:

(23) $$x_{0}\left(t\right)=B\cos\omega t$$

By employing the initial guess in Eq. (22) for k=0, we have the first-order solution as:

$$\begin{array}{cc} x_{1}\left(t\right) & =B\cos\omega t-\\ & \;-\frac{1}{\omega }E^{-1}\left(\frac{1}{v}E\left[\sin\omega t\right]E\left[\begin{array}{c} \left(1+Ad_{1}\cos\omega t+A^{2}d_{2}\cos^{2}\omega t+A^{3}d_{3}\cos^{3}\omega t\right)\left(-A^{2}\omega^{2}\cos\omega t\right)+d_{4}+Ad_{5}\cos\omega t\\ \;+A^{2}d_{6}\cos^{2}\omega t+A^{3}d_{7}\cos^{3}\omega t+A^{4}d_{8}\cos^{4}\omega t+A^{5}d_{9}\cos^{5}\omega t+A^{6}d_{10}\cos^{6}\omega t \end{array}\right]\right) \end{array}$$

Following relations can help to simplify the above equation:

$$\cos^{2n-1}A=\frac{1}{2^{2n-2}}\left\{\cos\left(2n-1\right)A+\left(\begin{array}{c} 2n-1\\ 1 \end{array}\right)\cos\left(2n-3\right)A+\ldots +\left(\begin{array}{c} 2n-1\\ n-1 \end{array}\right)\cos A\right\}$$

$$\cos^{2n}A=\frac{1}{2^{2n}}\left(\begin{array}{c} 2n\\ n \end{array}\right)+\frac{1}{2^{2n}-1}\left\{\cos2nA+\left(\begin{array}{c} 2n\\ 1 \end{array}\right)\cos\left(2n-2\right)A+\ldots +\left(\begin{array}{c} 2n\\ n-1 \end{array}\right)\cos2A\right\}$$

Consequently, we arrived at

(24) $$\begin{array}{ccc} x_{1}\left(t\right) & = & B\cos\omega t-\\ & & -\;\frac{1}{\omega }E^{-1}\left(\frac{1}{v}E\left[\sin\omega t\right]E\left[\Gamma_{0}+\Gamma_{1}\cos\omega t+\Gamma_{2}\cos2\omega t+\Gamma_{3}\cos3\omega t+\Gamma_{4}\cos4\omega t\;+\Gamma_{5}\cos5\omega t+\Gamma_{6}\cos6\omega t\right]\right) \end{array}$$

where the coefficients $\Gamma_{0},\Gamma_{1},\ldots ,\Gamma_{7}$ are given in Appendix B. To make Eq. (24) simpler, we can use the following formula.

(25) $$E^{-1}\left(\frac{1}{v}E\left[\sin\omega t\right]\;E\left[\cos\sigma \omega t\right]\right)=\left\{ \begin{array}{cc} 0.5t\sin\omega t,\; & \sigma =1\\ \frac{\cos\omega t-\cos\sigma \omega t}{\omega (\sigma^{2}-1)},\; & \sigma \neq 1 \end{array}\right.$$

Thus after simple calculations, we have

(26) $$\begin{array}{ccc} x_{1} & = & B\cos\omega t+\frac{\Gamma_{0}}{\omega^{2}}\left(\cos\omega t-1\right)-\frac{\Gamma_{1}}{2\omega }t\sin\omega t-\frac{\Gamma_{2}}{3\omega^{2}}\left(\cos\omega t-\cos2\omega t\right)\\ & & -\;\frac{\Gamma_{3}}{8\omega^{2}}\left(\cos\omega t-\cos3\omega t\right)-\frac{\Gamma_{4}}{15\omega^{2}}\left(\cos\omega t-\cos4\omega t\right)\\ & & -\;\frac{\Gamma_{5}}{24\omega^{2}}\left(\cos\omega t-\cos5\omega t\right)-\frac{\Gamma_{6}}{35\omega^{2}}\left(\cos\omega t-\cos6\omega t\right) \end{array}$$

The coefficient of tsinωt should be zero for the system to maintain its periodicity, therefore

(27) $$\frac{\Gamma_{1}}{2\omega }=0$$

or

$$-B\omega^{2}\left(1+\frac{3d_{2}B^{2}}{4}\right)+Bd_{5}+\frac{3d_{7}B^{3}}{4}+\frac{5d_{9}B^{5}}{8}=0$$

This equation yields the following nonlinear frequency-amplitude relationship:

(28) $$\omega =\sqrt{\frac{8d_{5}+6d_{7}B^{2}+5d_{9}B^{4}}{8+6d_{2}B^{2}}}$$

which corresponds to the first-order frequency provided by the SRHBM [21]. Consequently, the approximate first-order analytical solution is:

(29) $$x_{EVIM}=a_{0}+\left(a_{1}+B\right)\cos\omega t+a_{2}\cos2\omega t+a_{3}\cos3\omega t+a_{4}\cos4\omega t+a_{5}\cos5\omega t+a_{6}\cos6\omega t$$

where

(30) $$\begin{array}{ccc} a_{0} & = & -\frac{\Gamma_{0}}{\omega^{2}},\;\;a_{1}=\frac{1}{\omega^{2}}\left(\Gamma_{0}-\frac{\Gamma_{2}}{3}-\frac{\Gamma_{3}}{8}-\frac{\Gamma_{4}}{15}-\frac{\Gamma_{5}}{24}-\frac{\Gamma_{6}}{35}\right),\;\;a_{2}=\frac{\Gamma_{2}}{3\omega^{2}},\\ a_{3} & = & \frac{\Gamma_{3}}{8\omega^{2}},\;\;\;a_{4}=\frac{\Gamma_{4}}{15\omega^{2}},\;\;\;a_{5}=\frac{\Gamma_{5}}{24\omega^{2}},\;\;\;a_{6}=\frac{\Gamma_{6}}{35\omega^{2}} \end{array}$$

Therefore, using Eq. (28) and Eq. (29) respectively, it is possible to obtain the periodic property of the doubly clamped nanobeams-based N/MEMS caused by van der Waals forces.

## Results and discussion

To elucidate the reliability and accuracy of the obtained results by the variational iteration method, this section gives a comparison of the results with the exact ones and those in literature.

Table 1 evaluates the nonlinear frequencies produced by RK4 and EVIM (as determined by Eq. (28)) for various parameters. The last column's highest percentage error, which is less than 3%, demonstrates the high precision of the suggested technique. We can see that the van der Waals force parameter didn't affect the nonlinear frequency (see rows 5–7, 8, and 12). The increase in the van der Waals force results in no change in the amount of error, and the magnitude of the error is similar in this case. Thus we can say that this parameter causes the pull-in instability phenomena.

**Table 1:** Comparison of nonlinear frequencies gained by RK4 and EVIM.

α	B	λ	N	$\omega_{RK4}$	$\omega_{EVIM}$	% error
0.06	0.1	0.2	50	23.89044	23.30972	2.4308
0.06	0.2	0.2	50	23.89044	24.31427	1.7741
0.06	0.3	0.2	50	23.89044	24.32193	1.8061
0.06	0.4	0.2	50	23.89044	24.34699	1.9110
0.06	0.5	0.5	50	13.87017	14.00506	0.9725
0.06	0.5	1	50	13.87017	14.00506	0.9725
0.06	0.5	2	50	13.87017	14.00506	0.9725
0.1	0.1	0.1	10	13.87017	14.00506	0.9725
0.1	0.1	0.1	20	17.30905	17.17084	0.7985
0.1	0.1	0.1	30	20.07407	19.83769	1.1776
0.1	0.1	0.1	50	23.89044	24.30897	1.7519
0.1	0.1	1	10	13.87017	14.00506	0.9725
0.15	0.2	1.5	15	15.59103	15.67400	0.5322

**Fig. 2:**
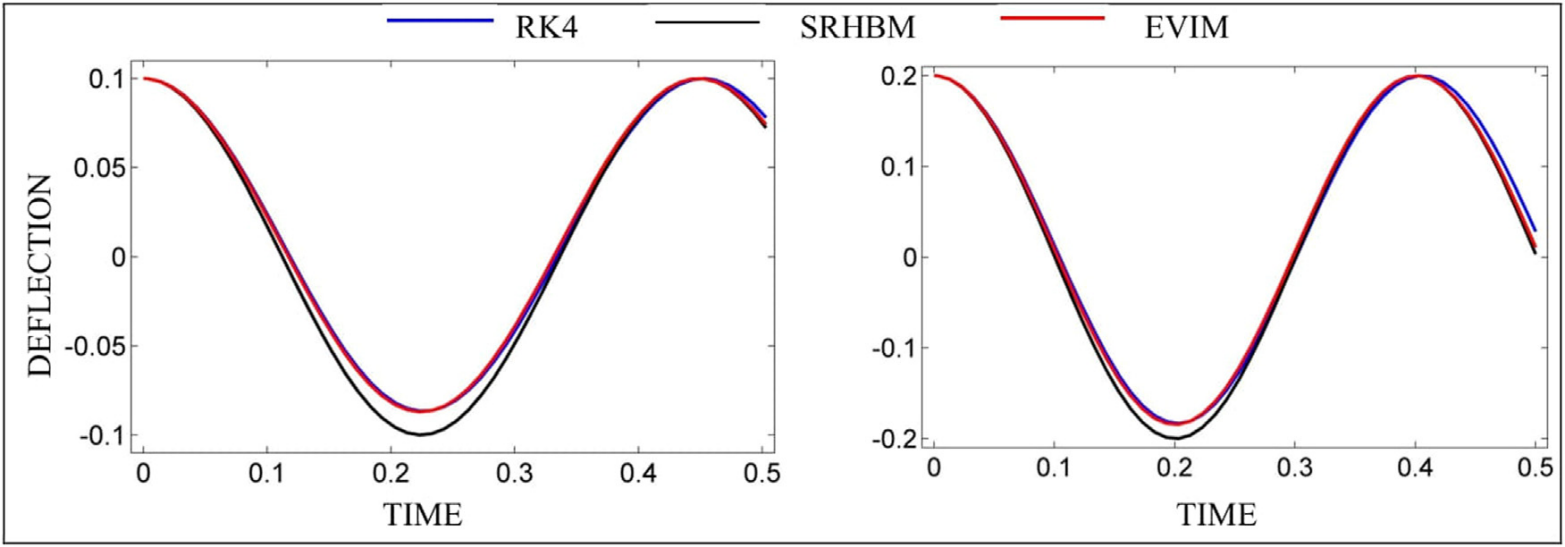
Comparison of mid-plane deflections and error estimations.

The effectiveness of the suggested approach can also be seen in Fig. 2. It demonstrates the comparison of the mid-plane deflection of nanobeam gained computationally from RK4 and attained analytically from EVIM and SRHBM [21], see Fig. 2, showing the reliability of the outcomes produced by the suggested method of EVIM. The reason for showing these graphs for S(0.1,10,0.11,1) on the left and T(0.2,15,0.145,1.45) on the right side of the panel is that the percentage errors are 0.97 and 0.53 (last two rows of Table 1) respectively, but the trough portion of the wave shows a clear difference between the RK4 and SRHBM solutions, whereas the EVIM solution closely matches that region. These results reveal the EVIM's enormous potential for tackling nonlinear problems over SRHBM.

**Fig. 3:**
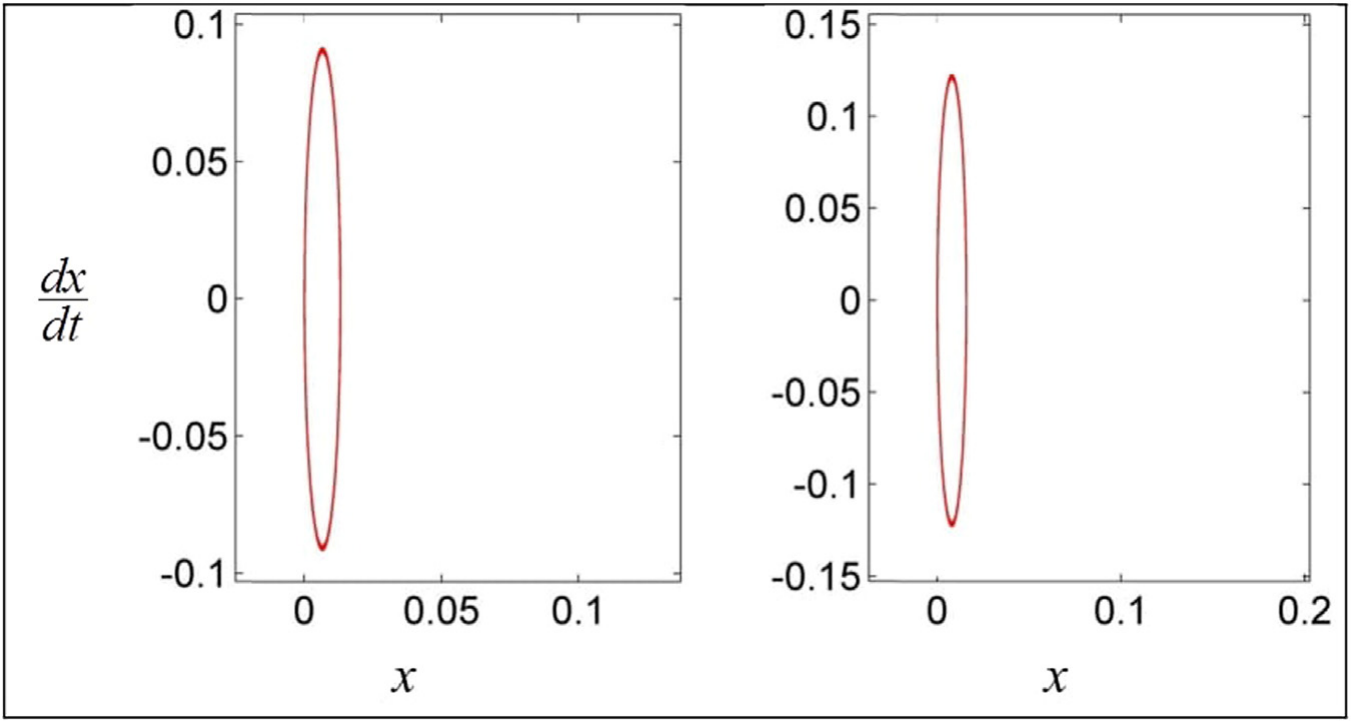
Phase diagram for various parameter values.

The phase portraits for the same set of parameter values are presented in Fig. 3. In basic oscillation theory, it is well-established that the closed orbits depicted in the phase diagrams are indicative of oscillatory behavior. It can be expected that there are critical values of the set of parameters on which pull-in solutions occur.

## Conclusion

The Euler-Bernoulli beam is considered in this study in a hunt for revealing the nonlinear oscillations of nanobeams. With the mid-plane stretching impact and van der Waals attractions taken into account, the mathematical model can remarkably be simple and effective. The nonlinear ordinary differential equation regulating the vibrations of nanobeams under clamped-clamped boundary constraints is then derived using the Galerkin approach. Combining the Elzaki transform with the variational iteration approach yields the nonlinear frequency. These techniques are then applied to get an analytical result for the nanobeam vibrations. When compared to the spreading residual harmonic balancing approach, a comparative analysis shows that the nonlinear frequency and analytical outcomes acquired by the aforementioned methods perform better.

We will extend our work for simply-supported and clamped-supported boundary conditions in near future. Furthermore, future research will focus on exploring a wide range of new applications for this technology, such as medical diagnostics, drug delivery, environmental monitoring, energy harvesting, security, and surveillance. These endeavors aim to enhance the technology's power and affordability.

## Declaration of Competing Interest

The authors declare that they have no known competing financial interests or personal relationships that could have appeared to influence the work reported in this paper.

## Data Availability

No data was used for the research described in the article.

## Appendix A

The following are the parameters that Eq. (18) defines.

$$h_{0}=\int_{0}^{1}\xi^{2}\;d\eta ,$$

$$h_{1}=-3\int_{0}^{1}\xi^{3}\;d\eta ,$$

$$h_{2}=3\int_{0}^{1}\xi^{4}\;d\eta ,$$

$$h_{3}=-\int_{0}^{1}\xi^{5}\;d\eta ,$$

$$h_{4}=-\lambda \int_{0}^{1}\xi \;d\eta ,$$

$$h_{5}=\int_{0}^{1}\left(\xi {\xi^{\prime \prime \prime }}^{\prime }\;-N\xi \xi^{\prime \prime }\right)\;d\eta ,$$

$$h_{6}=\int_{0}^{1}\left(-3\xi^{2}{\xi^{\prime \prime \prime }}^{\prime }+3N\xi^{2}\xi^{\prime \prime }\right)\;d\eta ,$$

$$h_{7}=\int_{0}^{1}\left(3\xi^{3}{\xi^{\prime \prime \prime }}^{\prime }\;-3N\xi^{3}\xi^{\prime \prime }\right)\;d\eta -\alpha \int_{0}^{1}\xi \xi^{\prime \prime }d\eta \int_{0}^{1}{\xi^{\prime }}^{2}d\eta ,$$

$$h_{8}=\int_{0}^{1}\left(-\xi^{4}{\xi^{\prime \prime \prime }}^{\prime }\;+N\xi^{4}\xi^{\prime \prime }\right)\;d\eta +3\alpha \int_{0}^{1}\xi^{2}\xi^{\prime \prime }d\eta \int_{0}^{1}{\xi^{\prime }}^{2}d\eta ,$$

$$h_{9}=-3\alpha \int_{0}^{1}\xi^{3}\xi^{\prime \prime }d\eta \int_{0}^{1}{\xi^{\prime }}^{2}d\eta ,$$

$$h_{10}=\alpha \int_{0}^{1}\xi^{4}\xi^{\prime \prime }d\eta \int_{0}^{1}{\xi^{\prime }}^{2}d\eta .$$

where ξ(η)=sinπη.

## Appendix B

Following are the coefficients for Eq. (24):

$$\Gamma_{0}=-B\omega^{2}\left(\frac{d_{1}B}{2}+\frac{3d_{3}B^{3}}{8}\right)+d_{4}+\frac{d_{6}B^{2}}{2}+\frac{3d_{8}B^{4}}{8}+\frac{5d_{10}B^{6}}{16},$$

$$\Gamma_{1}=-B\omega^{2}\left(1+\frac{3d_{2}B^{2}}{4}\right)+Bd_{5}+\frac{3d_{7}B^{3}}{4}+\frac{5d_{9}B^{5}}{8},$$

$$\Gamma_{2}=-B\omega^{2}\left(\frac{d_{1}B}{2}+\frac{d_{3}B^{3}}{2}\right)+\frac{d_{6}B^{2}}{2}+\frac{d_{8}B^{4}}{2}+\frac{15d_{10}B^{6}}{32},$$

$$\Gamma_{3}=-B\omega^{2}\left(\frac{d_{2}B^{2}}{4}\right)+\frac{d_{7}B^{3}}{4}+\frac{5d_{9}B^{5}}{16},$$

$$\Gamma_{4}=-B\omega^{2}\left(\frac{d_{3}B^{3}}{8}\right)+\frac{d_{8}B^{4}}{8}+\frac{3d_{10}B^{6}}{16},$$

$$\Gamma_{5}=\frac{d_{9}B^{5}}{16},$$

$$\Gamma_{6}=\frac{d_{10}B^{6}}{32}.$$
